# Reliability and Clinical Validity of the SARC-Global Questionnaire for Sarcopenia and Sarcopenic Obesity in Spanish Older Adults

**DOI:** 10.3390/nu17071206

**Published:** 2025-03-29

**Authors:** Juan Manuel Guardia-Baena, María del Carmen Carcelén-Fraile, Fidel Hita-Contreras, Agustín Aibar-Almazán, María de los Ángeles Arévalo-Ruíz, María Aurora Mesas-Aróstegui, Raquel Fábrega-Cuadros

**Affiliations:** 1 Department of Endocrinology and Nutrition, Virgen de las Nieves University Hospital, 18014 Granada, Spain; 2 Department of Educational Sciences, Faculty of Social Sciences, University of Atlántico Medio, 35017 Las Palmas de Gran Canaria, Spain; 3 Department of Health Sciences, Faculty of Health Sciences, University of Jaén, 23071 Jaén, Spain; 4 Department of Anaesthesiology, Virgen de las Nieves University Hospital, 18014 Granada, Spain; 5 Pediatrics Department, Hospital of Guadix, 18500 Granada, Spain

**Keywords:** SARC-Global, sensitivity, specificity, reliability, accuracy, sarcopenia, sarcopenic obesity, nutritional status, physical activity level

## Abstract

Background/Objectives: Sarcopenia and sarcopenic obesity (SO) are related to an increased risk of adverse outcomes. The objective of this study was to assess the internal and clinical validation of the Spanish version of the SARC-Global questionnaire, a sarcopenia risk screening tool, and its ability to detect sarcopenia, severe sarcopenia, and SO in adults aged ≥ 60 years. Methods: A total of 167 participants (73.22 ± 6.70 years, 71.26% women) completed the study. First, reliability was assessed by the inter-rater and the test–retest analyses. For the clinical validation, the risk of sarcopenia (SARC-Global) was compared to sarcopenia diagnosed using three operational definitions. The SARC-Global’s ability to detect severe sarcopenia (SS) and sarcopenic obesity assessed with body mass index (SO-BMI) and body fat percentage (SO-BFP), considering nutritional status and physical activity level, was also analyzed. Results: The Spanish SARC-Global questionnaire showed a substantial to excellent inter-rater and test–retest reliability. Regarding the clinical validation, sensitivity/specificity values to detect cases of sarcopenia were 85.71%/64.38% (EWGSOP2), 83.33%/65.81% (FNIH), and 54.55%/63.46% (AWGS-2019). Diagnostic accuracy ranged from 67.07% (FNIH) to 62.87% (AWGS-2019). The analysis also indicated that SARC-Global cutoff of 13.5 was the optimal score for severe sarcopenia (100.00% sensitivity and 80.49% specificity), SO-BMI (100% sensitivity and 80.49% specificity), and SO-PBF (80.00% sensitivity and 80.86% specificity). Conclusions: The Spanish version of the SARC-Global questionnaire is a reliable and clinically valid instrument for identifying people at the risk of sarcopenia, severe sarcopenia, and sarcopenic obesity in Spanish older adults.

## 1. Introduction

With age, a series of changes occurs in the body composition, such as a gradual decrease in lean mass and a progressive increase in body fat [1]. Rosenberg proposed the term sarcopenia in 1989 to describe this age-related decrease in muscle mass [2]. Since then, several working groups have proposed other definitions and diagnostic criteria for sarcopenia, using not only muscle mass, but also muscle strength and physical performance [3,4,5]. The prevalence of sarcopenia varies given the variety of definitions, cutoff values, and the instruments used to evaluate these parameters. According to a meta-analysis published in 2022 [6], the prevalence ranged from 8% to 36% in people aged < 60 years and from 10% to 27% in adults of 60 years and older. There are several study groups on sarcopenia that use different diagnosis criteria, cut-off points, and measurement instruments, such as the European Working Group on Sarcopenia in Older People (EWGSOP2) [7], the Asian Working Group on Sarcopenia (AWGS-2019) [3], or the Foundation for the National Institutes of Health (FNIH) Sarcopenia Project [5]. Sarcopenia has been formally recognized as a muscle disease by the International Classification of Disease (ICD-10, code M62.84) [8].

Sarcopenia has been linked to an increased risk of adverse outcomes, including cardiovascular diseases [9], mild cognitive decline [10], falls and fractures [11], physical disability [12], and frailty and mortality [7]. Therefore, sarcopenia is considered a major clinical problem for older people and public health [13]. Given the impact that sarcopenia has on older people, an effective screening and diagnostic tool that can be easily used in daily practice is of great importance.

The SARC-F has been recommended by the EWGSOP2 as a valid and consistent tool for identifying people at risk of sarcopenia and the associated adverse outcomes [7,14], and it has been used across different populations [15,16,17,18]. Although the SARC-F has shown high specificity, its sensitivity is relatively low, which makes this tool more effective for identifying people without the risk of sarcopenia, rather than those who have it [19,20]. In this context, the SARC-Global has been recently developed with the aim of increasing the sensitivity and accuracy in sarcopenia detection [21] by integrating additional clinical and anthropometric items. This questionnaire includes other variables, such as grip strength, sex, age, and the number of medications per day, and three anthropometric parameters that are important indicators of the nutritional status such as BMI, with the cutoffs described by Lipschitz (1994) [22] specifically for the elderly, and calf and arm circumferences, that can be used as a complementary tool for monitoring the nutritional status of elderly inpatients [23,24].

Low muscle strength and mass coexisting with obesity has been termed sarcopenic obesity (SO) [25], and its relationship is complex, with multiple interactions and factors implicated in the maintenance of muscle and fat mass [26]. When these two entities are combined, the health risks may be synergistically amplified. SO has been associated with increased functional disability, risk of fall, dependence, cognitive impairment, coronary artery disease, or dyslipidemia, has been considered a poor prognostic factor in cancer, and is related to greater morbidity and mortality [27,28,29].

Several lifestyle factors are related to a high risk for sarcopenia and SO. Older persons are at higher risk of a poor nutritional status, which is an important factor involved in the etiology of body composition abnormalities [30,31]. On the other hand, muscle strength and mass have been found to be significantly higher in people engaged in moderate to vigorous physical exercise than in those who did not exercise [32], and a positive association between smoking habits and sarcopenia has been described [33].

To the extent of our knowledge, the psychometric properties of the Spanish version of the SARC-Global have not yet been examined. The objective of the present study was to analyze the reliability and clinical validation of the SARC-Global questionnaire, and to assess its ability to detect patients with sarcopenia, severe sarcopenia, and SO in Spanish adults aged 60 years and older. We hypothesized that the Spanish SARC-Global questionnaire is a reliable and clinically valid tool for identifying people at the risk of sarcopenia, severe sarcopenia, and SO, taking into account some other possible risk factors such as nutritional status, physical activity level, or a smoking habit.

## 2. Materials and Methods

### 2.1. Study Design and Participants

A cross-sectional study was performed from December 2024 to February 2025. Participants were recruited from several day centers for older adults in Jaén (Spain). Participants were contacted by e-mail, local media, and social networks. From the 180 volunteers who were initially contacted, 167 finally participated in the present validation study. This sample size is considered appropriate in accordance with the guidelines of the Sarcopenia Special Interest Group of the European Geriatric Medicine Society (EuGMS) [34] and with the psychometric recommendations described by Kline (at least 100 participants) [35]. The inclusion criteria were as follows: people aged 60 years or older, Spanish native speakers, living independently, being able to walk independently or with aids (ambulatory), understanding the purpose of this study, and providing their consent to complete the questionnaires.

Participants were excluded if bioelectrical impedance analysis (BIA) was contraindicated (cardiac pacemaker, metal implants), they were bed-bound, had severe or chronic conditions that could interfere with their answers, and have not provided their willingness to take part in this study. This study was performed following the ethical principles of the Declaration of Helsinki, and each participant provided a written informed consent before the beginning of the study.

### 2.2. Procedure

Following the guidelines described by the World Health Organization (WHO) methodology for the translation and intercultural adaptation of health questionnaires [36], and the recommendations of the Sarcopenia Special Interest Group of the EuGMS, the procedure consisted of two phases [34].

First, we performed the translation and cross-cultural adaptation of the SARC-Global into Spanish. Initially, two bilingual experts, together with two clinical professionals with experience in this topic, provided a preliminary Spanish version, which was filled out by ten participants (50% women) in order to make sure that the items and instructions of the questionnaire were understood. After this, the Spanish version of the SARC-Global was translated back into English, and both the Spanish and English versions were compared. Finally, in this first phase, inter-rater and test–retest reliability were determined. Inter-rater reliability was evaluated by two independent experts on 20 participants (10 men and 10 women). The test–retest reliability was determined by two independent researchers on a sample of 28 participants (50% women), who completed the questionnaire again after a 2-week period. For the clinical validation of the Spanish SARC-Global, we compared the questionnaire to the presence of sarcopenia, severe sarcopenia, and SO.

### 2.3. Outcomes

#### 2.3.1. SARC-Global Questionnaire

The SARC-Global consists of 12 items, which refer to strength, assistance for walking, getting up from a chair, climbing stairs, falls, sex, age, medications, body mass index (BMI), handgrip strength, arm circumference, and calf circumference. These items provide a total score which ranges from 0 to 26 points, with higher scores indicating a greater risk of sarcopenia. SARC-Global scores ≥ 11 points indicate the risk of sarcopenia [37]. Besides age and sex, other demographic information such as the educational level, marital and occupational status, and the existence of a smoking habit were collected.

#### 2.3.2. Sarcopenia

Handgrip strength was assessed with a handgrip dynamometer (TKK 5001, Grip-A, Takei, Tokyo, Japan). Calf circumference was measured at the widest perimeter of the right leg calf with the participant in a standing position [38]. Arm circumference was assessed in the midpoint between the acromion of the scapula and the olecranon process of the ulna, while the participants were sitting comfortably. Both circumferences were obtained with a flexible and non-extensible measuring tape (SECA 201, Seca, Ltd., Hamburg, Germany), and expressed in centimeters, with an accuracy of 0.1 cm. The two measurements were performed twice and the mean was employed for the analysis.

Sarcopenia was diagnosed as low handgrip strength together with low muscle mass, following the EWGSOP2 recommendations [7]. Low muscle strength, as assessed by handgrip strength, was established by using the previously described cutoff points, <16 kg (women) and <27 kg (men). Muscle mass, as assessed by appendicular skeletal muscle mass index (ASMI), was obtained by BIA (InBody 720, Biospace Co., Ltd.; Seoul, Republic of Korea). ASMI was calculated by dividing the appendicular skeletal muscle mass by the height in square meters (kg/m^2^), where low muscle mass was defined as <5.5 kg/m^2^ and <7 kg/m^2^ for women and men, respectively. Gait speed was evaluated by the TUG test by employing this equation: [6/(TUG time) × 1.62] [39]. Low gait speed, determined by the following cutoffs: ≤0.8 m/s, together with low muscle strength and mass, indicated severe sarcopenia [7].

When the AWGS-2019 criteria were followed, the cutoffs for low muscle strength were <27 kg (men) and <18 kg (women), for muscle mass < 7 kg/m^2^ (men) and <5.7 kg/m^2^ (women), and low gait speed was determined by ≤1.0 m/s (both men and women). Sarcopenia (AWGS-2019) was determined as low muscle mass together with low muscle strength or slow gait speed [3]. Finally, according to the FNIH, ASMI was obtained by using the BMI instead of the squared height, with cutoffs of <0.79 and <0.51 (for men and women, respectively), while for muscle strength they were <26 kg and <16 kg (men and women, respectively), and for gait speed it was ≤0.8 m/s (men and women). Sarcopenia (FNIH) was diagnosed as low muscle mass and strength, together with a slow gait speed [5].

#### 2.3.3. Sarcopenic Obesity

The body mass index (BMI) was calculated as weight (kg) divided by height (m^2^) [40]. An adult height scale (T201-T4 Asimed, Barcelona, Spain) was employed to assess height, and a 100 g to 130 kg precision digital weight scale (Tefal, Barcelona, Spain) was used for weight status. The percentage of body fat (PBF) was obtained by BIA. Obesity was defined as BMI values ≥ 30 kg/m^2^ [40], and a cutoff value of ≥27% (men) and ≥38% (women) was used to determine obesity with PBF [41]. SO was defined as the presence of sarcopenia (EWGSOP2) together with obesity as assessed by either the BMI or the PBF.

#### 2.3.4. Nutritional Status

The nutritional status of the participants was assessed by the Mini Nutritional Assessment (MNA) questionnaire [42]. This tool consists of 18 items which provide four categories (anthropometric, dietary, global and subjective assessment) and a total score (maximum of 30 points). Greater scores indicate a better nutritional status. An MNA score ≥ 24 indicates adequate nutritional status, between 17 and 23.5 risk of malnutrition, and <17 protein–calorie malnutrition.

#### 2.3.5. Physical Activity Level

The International Physical Activity Questionnaire-Short Form (IPAQ-SF) was employed to evaluate the physical activity level [43]. It has seven items that requests information on physical activity during usual week with 4 intensity levels (vigorous-intensity activity, moderate-intensity activity, walking, and sitting). The physical activity level was calculated and represented as a metabolic equivalent (MET)-minute/week.

### 2.4. Statistical Analysis

The Kolmogorov–Smirnov test was used to evaluate the normality of the distribution of continuous variables. Student’s *t*- and Chi-square tests were used for comparisons between continuous (described as mean and standard deviation) and categorical variables (described as frequency and percentage), respectively. Cronbach’s α coefficient was used to assess internal consistency. Regarding the inter-rater and test–retest reliability, we employed the intraclass correlation coefficient by Shrout and Fleiss (ICC_2,1_). Reliability was classified as excellent if ICC > 0.90, substantial between 0.75 and 0.90, moderate between 0.40 and 0.75, and poor when ICC < 0.40 [44]. With regards of the clinical validation, we determined the sensitivity, specificity, positive predictive value, negative predictive value, and accuracy of the Spanish SARC-Global (cutoff ≥ 11) in screening sarcopenia, according to the diagnosis criteria described by the EWGSOP2 [7], AWGS-2019 [3], and FINH [5]. A Multivariate logistic regression analysis was used to determine the independent associations between the SARC-Global score and the presence of severe sarcopenia, and SO. Nutritional status, physical activity level, and a smoking habit were considered as possible confounders. The area under the curve (AUC) based on the receiver-operating characteristics (ROC) analysis was calculated to determine the ability of the SARC-Global score to discriminate between the participants with and without severe sarcopenia and SO, as well as the Youden index (J), which was considered high when ≥0.6 and low when 0.6 [45]. The level of statistical significance was set at a *p* value ≤ 0.05. The SPSS 20.0 statistical package (SPSS, Inc., Chicago, IL, USA) was employed for data management and statistical analysis.

## 3. Results

The descriptive characteristics of the sample are shown in Table 1. A total of 167 people participated (71.6% women) with a mean age of 73.22 ± 6.70 years. The majority of participants were retired (85.63%), married or living with a partner (52.69%), had a primary or no education (58.68%), and were non-smokers (94.71%). The mean SARC-Global total score questionnaire was 9.25 ± 4.22, which is below the risk limit for sarcopenia, and 63 participants (37.72%) were sarcopenic as assessed by the SARC-Global. When the possible differences between these variables were analyzed with the consideration of the possibility of having sarcopenia according to the SARC-Global (mean score of 9.25 ± 4.22), it was observed that, as expected, older participants presented a significantly higher risk of sarcopenia (*p* < 0.001), as well as those who were retired (*p* = 0.001) and those with primary studies or less (*p* = 0.09). As for the nutritional status, the mean MNA score for all the participants was 26.16 ± 2.21, which indicates adequate nutrition. With regard to MNA groups, only 14.97% were at risk of malnutrition and no one was categorized as protein–calorie malnourished. The participants at risk of sarcopenia (SARC-Global) showed a significantly lower mean score in the MNA (*p* = 0.001).

The analysis of the psychometric properties revealed that the inter-rater reliability for the SARC-Global total score was excellent (0.971, 95% CI: 0.93–0.99). Regarding test–retest reliability (Table 2), the SARC-Global was consistent across time. The ICC for the total score of the SARC-Global was 0.919 (95% CI: 0.82–0.96) which means excellent reliability, while for the items, test–retest reliability ranged from substantial (items 2, 3, 4, 5, and 9) to excellent (items 1, 6, 7, 8, 10, 11 and 12), with ICC values varying from 0.780 (item 2) to 1.000 (items 6 and 7). As for the internal consistency, the Cronbach’s α value was 0.601.

As for the prevalence of sarcopenia, 4.19% of the participants presented it when following the EWGSOP2 criteria, while 6.59% and 7.19% were observed according to AGWS-2019 and FNIH, respectively. In order to assess the clinical validation. Table 3 displays that the sensitivity of the SARC-Global to detect cases of sarcopenia varied from 85.71% (EWGSOP2) to 54.55% (AWGS-2019), while the specificity was similar under all the diagnostic criteria (from 63.46% with AWGS-2019 to 65.81% with FNIH). The diagnostic accuracy of the SARC-Global for sarcopenia ranged from 62.87% (AWGS-2019) to 67.07% (FNIH).

The independent associations between the groups with and without the risk of sarcopenia according to the SARC-Global and the prevalence of severe sarcopenia (1.80%), SO-BMI (1.80%), and SO-PGC (2.99%) were analyzed considering nutritional status, physical activity level, and smoking habits as possible confounders. The results of the multivariate analysis (Table 4) showed a significantly higher SARC-Global total score in participants with severe sarcopenia (15.33 ± 1.15 vs. 9.14 ± 4.28, *p* = 0.050), SO-BMI (15.67 ± 1.53 vs. 9.13 ± 4.27, *p* = 0.021), and SO-PGC (14.20 ± 3.63 vs. 9.10 ± 4.26, *p* = 0.023).

**Figure 1:**
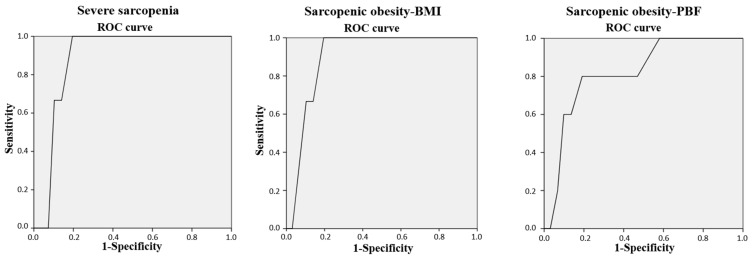
The ROC curve of the SARC-Global total score for discriminating between participants with severe sarcopenia, sarcopenic obesity–BMI, and sarcopenic obesity–PBF. BMI: body mass index. PBF: percentage of body fat. ROC: Receiver Operating Characteristic.

The results of the ROC curve analysis (Figure 1) showed that the SARC-Global score was able to discriminate between participants with or without severe sarcopenia, SO-BMI, and SO-PBF, with AUCs values of 0.89 (95% CI: 0.82–0.95, *p* = 0.022), 0.90 (95% CI: 0.83–0.97, *p* = 0.018), and 0.82 (95% CI: 0.66–0.98, *p* = 0.015), respectively. The analysis determined that a SARC-Global total score of 13.5 was the optimal score for severe sarcopenia (100.00% sensitivity and 80.49% specificity, J = 0.80), SO-BMI (100% sensitivity and 80.49% specificity, J = 0.81), and SO-PBF (80.00% sensitivity and 80.86% specificity, J = 0.80).

## 4. Discussion

The objective of the present work was to analyze the internal and clinical validation of the SARC-Global in Spanish older adults aged 60 years and older. The results indicate that this questionnaire is a reliable and valid tool, and is able to detect not only sarcopenia, but also severe sarcopenia and sarcopenic obesity, in this population.

The aging of the world population is a fact, and is a result of the ongoing development and the consequent rise in life expectancy, as well as the decline of the birth rate. It is estimated that between 2015 and 2050, the percentage of the world’s population aged >60 years will almost double, going from 12% to 22% [46]. For this reason, the management and prevention of age-related diseases is increasingly important.

Measurement instruments play an important role in research, clinical practice and health assessment [47]. Reliability and validity are two of the most important and fundamental aspects in the assessment of any measuring methodology [48]. Reliability is a psychometric property that assesses the reproducibility of a test or other measurement in repeated trials performed on the same participants. A higher reliability indicates a small measurement error [49]. In order to evaluate the test–retest reliability, the Spanish version of the SARC-Global was administered again to a subsample (*n* = 28, 50% women) two weeks later, following the guidelines described by the EUGMS for the cross-cultural adaptation and validation of the SARC-F questionnaire [34]. This period of time is long enough for the participants to forget their previous answers but short enough to prevent changes in their physical capacity that could alter their answers [50]. Our results indicated excellent test–retest reliability for the SARC-Global total score (ICC_2,1_ > 0.90), while the results for every single item varied from substantial to excellent. As expected, the responses for items 6 and 7 were identical, since they refer to gender and age. Similarly, inter-rater reliability was excellent for the total score. The Cronbach’s α coefficient obtained in this study was 0.601, which is similar to the 0.63 described by Lopes et al. (2025) in the original validation of the questionnaire [21], suggesting moderate but acceptable internal consistency [51]. Given that this is a multidimensional tool, this could be explained by the inclusion of items that provide diverse information (such as anthropometric, demographic, or functional variables).

The SARC-F is a validated tool for screening sarcopenia, but its sensitivity is relatively low, which would make it more effective for determining the absence of sarcopenia. This has also happened with the evolution of the questionnaire. For instance, Krzymińska-Siemaszko et al. (2020) [52], in a study performed on community-dwelling older adults from Poland, found that, using the EWGSOP2 criteria, the sensitivity of SARC-F, SARC-CalF (with a 31 cm and 33/34 cm), and SARC-F + EBM (SARC-F + age and BMI) were 37.5%, 37.5%, 62.5%, and 55.0%, respectively, while the specificity varied from 85.9%, 93.9%, 86.9%, and 70.7%, respectively. When the diagnostic values of the SARC-F were analyzed under the different diagnostic criteria, the results were similar. For example, sensitivity values of 50% (EWGSOP2), 47.4% (FNIH), and 39.1% (AWGS) were observed with the Polish version [53], while Boteta-Gomes et al. (2024) [18] found 20% (EWGSOP2) and 28.6% (AWGS-2019) in the Portuguese validation.

Lopes et al. (2025), who developed the SARC-Global, found that the sensitivity of the questionnaire to detect sarcopenia (EWGSOP2) was 74%, higher than that observed with the SARC-F (21%) and with the SARC-Calf (34%), while the specificity was lower (0.75, 0.83 and 0.92, respectively) [21]. In our analysis, the SARC-Global showed a higher sensitivity in sarcopenia detection when the EWGSOP2 criteria were used (85.71%), while the specificity was lower (64.38%). On the other hand, the prevalence impacted both PPV and NPV values, and as the prevalence decreased, the PPV decreased, while the NPV increased. Our results showed very high NPV values, but also a low PPV for sarcopenia detection, which could be explained by the prevalence observed in the present study (4.19% according the EWGSOP2). This supports the utility of the questionnaire as a screening tool in low-prevalence settings by ruling out sarcopenia (high NPV).

Besides age-related muscle loss, several lifestyle factors have been associated with sarcopenia. Among many other harmful effects, chronic smoking can lead to widespread muscle loss and result in sarcopenia [33]. Current moderate smokers are more likely to have sarcopenia without obesity than those who have never smoked, while a greater number of cigarettes per day is associated with a greater likelihood of having SO [54].

Older persons are at higher risk of poor nutritional status [30], and it has been described that, in older people with malnutrition, the risk of developing sarcopenia is fourteen times greater than in those with a normal nutritional status [55]. Murawiak et al. (2022) demonstrated that a poor nutritional status (assessed with the MNA questionnaire) was present in over 80% of the participants with sarcopenia in their study [31]. On the other hand, Itani et al. (2024) showed that, in adults aged 60 years and over, the risk of having a slow gait speed, an indicator of severe sarcopenia, was 75% lower in those with a higher dietary adequacy score (MNA) [56], and Maccarone et al. (2023) reported that participants with severe sarcopenia had significantly lower values of MNA and BMI compared to those affected by probable or confirmed sarcopenia [57]. As for SO, Chang et al. (2020) concluded that central obesity and sarcopenia were interactively associated with the nutritional status of people aged ≥ 65 years old living in a rural community [58], and it has been described that people with SO have a higher risk of malnutrition compared to those with obesity alone [31]. A recent review highlights how a poor dietary quality, especially a high consumption of ultra-processed foods, correlates with reduced muscle mass, increased fat accumulation, and metabolic dysfunctions that may predispose individuals at any age to sarcopenia and SO. Therefore, it is of great importance to integrate nutritional interventions in the management and prevention strategies [59].

Regarding the level of physical activity and sedentary lifestyle habits, it has been demonstrated that, in older adults, sedentary behavior is independently positively associated with sarcopenia, especially in women [60,61]. In fact, an increase in moderate to vigorous physical activities, replacing sedentary lifestyle habits and light physical activity, is associated with a reduction in the prevalence of sarcopenia [62]. The practice of exercise programs either alone or together with dietary supplements improves muscle-related parameters and reduces fat-related outcomes in older adults with SO [63].

Slow gait speed has been associated with adverse outcomes in older adults, such as cardiovascular and respiratory disease or worse cognitive function [6,64], as well as with a loss of independence in the performance of activities of daily living [65], especially when combined with sarcopenia. Furthermore, gait speed has been shown to be the component of physical function most associated with sarcopenia and frailty, and has been identified as a significant predictor of frailty and all-cause mortality [66,67]. According to the EWGSOP2, a single cut-off speed ≤ 0.8 m/s (together with low muscle strength and quantity/quality) is advised as an indicator of severe sarcopenia [7]. Therefore, it is also very important to assess the risk of severe sarcopenia cases.

The results of the multivariate analysis showed that, regardless of the nutritional status, physical activity level, and the presence of a smoking habit, a SARC-Global score was positively associated with the presence of severe sarcopenia. Moreover, the analysis of the ROC curve showed that a cutoff point of 13.5 in the SARC-Global was the optimal score to detect cases of severe sarcopenia with a high Youden index (≥0.6).

Sarcopenic obesity is a prevalent disease with negative clinical consequences of great impact, since it has been shown to be a strong and independent risk factor for, among others, frailty, a greater number of comorbidities and mortality, especially in older people [68,69]. In addition to all this, the detection of SO is very important for the design of therapeutic interventions, because diets aimed at decreasing body weight can also lead to a decrease in muscle mass [31]. As with severe sarcopenia, multivariate analysis indicated that SARC-Global was independently associated with SO assessed by BMI and PGC, taking into account nutritional status, level of physical activity, and smoking as confounding variables. When the ROC curve was analyzed, the best cutoff for SO assessed with BMI and PBF was also 13.5, with high sensitivity, specificity, and Youden index values. Therefore, in our sample, the Spanish SARC-Global has been shown to be a good tool for screening sarcopenic obesity.

Some limitations must be acknowledged. This study was carried out on 167 community-dwelling adults aged ≥ 60 years, but, for example, institutionalized people were not considered. Furthermore, the percentage of women was higher than that of men (71.6%), and the participants belonged to two cities from a specific region of Spain. The evaluation of body composition was carried out using BIA, which, although it is a validated and recommended instrument [7], requires specific conditions, or, for instance, can be influenced by the hydration status of the patient. We recommend that future studies should be performed on a larger, more general sample, including institutionalized or hospitalized elderly individuals from different geographical regions.

## 5. Conclusions

The SARC-Global questionnaire was successfully adapted in Spanish community-dwelling adults aged ≥ 60 years. The assessment of the psychometric properties showed excellent inter-rater reliability and substantial to excellent test–retest reliability. As for the clinical validation, the SARC-Global showed high sensitivity and appropriate specificity values to detect sarcopenia. Moreover, our results indicated that a cutoff of 13.5 could discriminate between participants with or without severe sarcopenia and sarcopenic obesity with high sensitivity and specificity.

## Figures and Tables

**Table 1:** Descriptive characteristics of the participants and the differences according to the SARC-Global score.

		All the Participants (*n* = 167)		SARC-Global				*p*-Value
				Non Sarcopenic (*n* = 104)		Sarcopenic (*n* = 63)		
Age (years) ^a^		73.22	6.70	70.61	5.31	77.52	6.55	<0.001
Gender ^b^	Men	48	28.74	25	24.04	23	36.51	0.084
	Women	119	71.26	79	75.96	40	63.49	
Ocupation ^b^	Retired	143	85.63	81	77.88	62	98.41	0.001
	Active Worker	4	2.40	4	3.85	0	0.00	
	Unemployed	20	11.98	19	18.27	1	1.59	
Marital status ^b^	Single	14	8.38	12	11.54	2	3.17	0.167
	Married	88	52.69	53	50.96	35	55.56	
	Separated/divorced/widowed	65	38.92	39	37.50	26	41.27	
Education ^b^	Primary or less	98	58.68	53	50.96	45.00	71.43	0.009
	Secondary o higher	69	41.32	51	49.04	18.00	28.57	
Smoker ^b^	No	157	94.01	96	92.31	61	96.83	0.233
	Yes	10	5.99	8	7.69	2	3.17	
MNA score ^a^		26.16	2.21	26.61	2.00	25.43	2.36	0.001
IPAQ score (MET-min/week) ^a^		1234.94	1084.72	1340.08	1092.17	1061.39	1058.08	0.108

IPAQ: international physical activity questionnaire. MET: metabolic equivalent. MNA: mini nutritional assessment. ^a^ Data presented as means and standard deviations. ^b^ Data presented as frequencies and percentages.

**Table 2:** Test–retest reliability of the SARC-Global questionnaire (*n* = 28).

	ICC	95% CI		*p*-Value	Reliability
Item 1	0.97	0.93	0.98	<0.001	Excellent
Item 2	0.78	0.53	0.90	<0.001	Substantial
Item 3	0.79	0.56	0.90	<0.001	Substantial
Item 4	0.90	0.78	0.95	<0.001	Substantial
Item 5	0.90	0.78	0.95	<0.001	Substantial
Item 6	10.00	-	-	-	Excellent
Item 7	10.00	-	-	-	Excellent
Item 8	0.93	0.84	0.97	<0.001	Excellent
Item 9	0.90	0.78	0.95	<0.001	Substantial
Item 10	0.93	0.84	0.97	<0.001	Excellent
Item 11	0.96	0.92	0.98	<0.001	Excellent
Item 12	0.96	0.91	0.98	<0.001	Excellent
Total score	0.92	0.82	0.96	<0.001	Excellent

CI: confidence interval. ICC: intraclass correlation coefficient.

**Table 3:** Diagnostic values of the SARC-Global for sarcopenia according to different diagnostic criteria.

	Sarcopenia SARC-GLOBAL					
	Sensitivity (%)	Specificity (%)	PPV (%)	NPV (%)	Accuracy	AUC
Sarcopenia EWGSOP2	85.71	64.38	9.52	99.04	65.27	0.825
Sarcopenia AWGS-2019	54.55	63.46	9.52	95.19	62.87	0.842
Sarcopenia FNIH	83.33	65.81	15.87	98.08	67.07	0.676

AUC: area under the curve. AWGS-2019: Asian Working Group on Sarcopenia-2019. EWGSOP2: European Working Group on Sarcopenia in Older People-revised. FNIH: Foundation for the National Institutes of Health. PPV: positive predictive value. NPV: negative predictive value.

**Table 4:** Multivariate logistic regression analyses for sarcopenia, severe sarcopenia, and sarcopenic obesity.

		Exp (B)	95% CI		*p*-Value
			Inferior	Superior	
Severe sarcopenia	SARC-Global score	1.48	1.00	2.19	0.050
Sarcopenic obesity-BMI	SARC-Global score	1.55	1.01	2.37	0.021
Sarcopenic obesity-PBF	SARC-Global score	1.34	1.04	1.71	0.023

CI: Confidence Interval. Exp (B): odds ratio.

## Data Availability

The original contributions presented in this study are included in the article. Further inquiries can be directed to the corresponding author.
